# On the Recent Advances in the Theory of Vision

**Published:** 1872-07

**Authors:** H. Helmholtz

**Affiliations:** The Royal Society of London


ART. IV.—*On the recent Advances in the Theory of Vision. By* H. Helmholtz, (*of the Royal Society of London.*)

Translated from *Les Annales d' Oculstique*, By F. W. Abbott, M. D.

(*Continued.*)

### III. Visual Perceptions.

Colors, which engaged our attention in the preceding chapter, are among the most beautiful ornaments of nature, and they also aid us in distinguishing and recognizing objects; but the perception of them is much less important than that of *dimensions in space*, a perception which the eye furnishes to us with a rapidity and a variety of conditions which no other sense can rival. In appreciations of this kind, touch gives us, without doubt, more complete indications; it instructs us immediately as to the resistance, mass and weight. But, upon the one hand, touch can be exercised only where we are able to reach, and on the other hand, where we have to do with minute quantities, its appreciations are far from being as fine as those of which sight is capable. Nevertheless, as is demonstrated by observations made upon those born blind, touch is sufficient to develop in us the notions of space. We may even convince ourselves that the touch serves us constantly in controlling and correcting, if need be, the notions of space furnished by the eye, and that we always refer, as a last resort, to the information furnished by touch. Sight and touch, these two senses so diversely endowed, complement each other in the happiest manner in the accomplishment of their common task. While touch cannot overleap the narrow horizon by which it is bounded, sight stretches away to infinity, rivaling the flight of the boldest imagination.

This relation is of the greatest importance for the subject which now engages our attention. In fact, we have here to do only with the sense of sight; since that of touch suffices to completely develop in man the notions of space we may at once consider these notions as pre-existing essentially, and confine ourselves to seeking out how the concordance between the notions of space which are given us by sight and by touch is produced. We will reserve, to consider in the last place, the question of how sensual perceptions can transform themselves into notions of space.

However little attention we give it, we recognize first of all that the perception of impressions by different nervous apparatus is far from involving necessarily the notion of causes separated in space. Hence it is that our senses may perceive, in the same room, the light, the heat, the different sounds of a hamony and an odor, and that for all that, we recognize the simultaneous presence of all these agents in the air of the room.

So also, a composite color excites upon our retina three elementary sensations which we do not separate, although they probably reside indifferent nerves. A cord of a musical instrument, a human voice, emit simultaneously a fundamental sound and a series of hamonics which probably call into action as many different nerves, and the sensation remains none the less undivided. Finally, a mass of aliments affect differently the different parts of the tongue and act upon our smell at the moment of deglutition, nevertheless we generally unite the impressions which attain these different nervous systems in a single one which we call the taste of these aliments. A very little attention suffices to recognize by what parts of our body these different impressions reach us; but there does not result from this the necessity of localizing in different points of space the object which causes these sensations in us.

In the way in which vision is accomplished we find a fact of this kind; this is binocular vision, which is single, in spite of the separation of the impressions received upon the two retinas. We understand that this is a particular case of a much more general law.

If therefore the retina receives an image of the objects which fill the visual field, and if the different parts of this image excite different fibres, still this does not constitute a sufficient reason to attribute these sensations to parts differently situated in the visual field. There is evidently wanting the intervention of something which would lead us to the notion that these impressions correspond to different points of space.

This same problem is evidently propounded to us in an analogous manner with respect to touch. When the skin is simultaneously touched at two places, there results the irritation of two sensitive nerve fibres. But the separation of these fibres is not sufficient in itself for us to recognize that we have been touched in two different points and by two different objects. Our judgment may depend upon accessory circumstances in addition. When we place a finger of each hand upon the table, if we feel a grain of sand under both of these fingers, there is formed in us the notion of two grains of sand. If now we put both fingers together, and interpose a grain of sand, it may be that this grain will be felt by the very same two fibres as before, nevertheless the perception is reduced to that of one single grain. It is plain that here the simultaneous cognizance of the position of our members exercises its influence upon the notion which we form, and we know that when we form a false or incomplete idea of the position of our fingers, when they are crossed for example, we think that we feel two objects when in truth there is only one.

What then is the element which adds its effects to those of the state ot isolation of the sensitive nerves, and which gives us a correct notion of the separation of objects in space ? We here fall into a discussion which is not yet finished. Some respond, with Jean Muller, that the sensitive organ, retina or skin, perceives itself in space in consequence of an innate notion, and that we transport the received impressions to the place where we perceive the corresponding part of the organ. We will designate this opinion under the name of the *naturalistic theory* of the notions of space. It cuts short all research relating to the origin of these notions, since it considers them as something original, innate, as not susceptable of explanation.

The rival opinion has already been expressed in a general way by the English sensualists, such as, Malineux, J Locke, Jurine. Its application to the theory of vision could only be made very recently since it had to be preceded, among other things, by the study of the movements of the eye. It is especially owing to the discovery of the stereoscope by Wheatstone, that the difficulties and the contradictions of the naturalistic became apparent; a solution more in conformity with the ideas of the sensualists was then sought for, and to this we will give the name of the *emperical* theory of vision. This theory supposes that our sensations give us, as it relates to objects and external phenomena, only signs whose signification becomes familiar to us by experience and habit. In accordance with this theory, the perception of position in space is taught at first by the movements which we execute; that of the positions in the field of vision is attributable particularly to the movements of the eyes. It is clear that, just as is the case with the naturalistic theory, the empirical theory must suppose, between the sensations furnished by the different points of the retina, some peculiar differences attributable only to their difference of position; in truth if it were not so, it would be impossible to explain the localizations in the field of vision. It is necessary that the sensation of red which affects the retina, on the right should differ in some way from that of the red which affects this same retina on the left, and the difference between these two sensations of red must be other than the difference between two sensations of red, unequal, yet experienced successively at the same point of the retina. We will call with Lotze, *local sign* of the sensation, this difference which the same color produces at different points of the retina, and this without our knowing how to define, as yet, the nature of this difference. I consider it premature to construct any hypotheses whatever as to the nature of these local signs; it suffices for us to say that their existence is an irrefragable consequence of the perception of local differences in the field of vision.

Such, then, is the difference between the two opinions before us, The emperical theory considers the local signs as simply signs, their nature matters little to it; it admits that the significance of its signs, as it relates to the perception of the external world, can be and really is the result of experience. It becomes useless then to pre-suppose any accord whatever between the local signs and the local differences which correspond to them. According to the naturalistic theory, on the contrary, the local signs would be nothing less than immediate notions of the local differences, both of their nature and of their size. We see then that we are here engaged in the contest between two phylosophical systems, one of which supposes a pre-established harmony between the laws of thought and those of the external world, and the other seeks to deduce from experience all the conformity which may exist between the external world and our ideas.

So long as we limit ourselves to considering a superficial field, whose different parts are not apparently at a different distance from the eye; so long as we look, for example, only at the sky and the distant parts of the country, both theories account almost equally well for the perception of dimensions in this field. The image formed upon the retinal surface corresponds to the superficial image of which we acquire the notion. The disagreements are not of a sufficiently grave nature as not to be reconcilable with the naturalistic theory, by means of relatively simple explanations or hypotheses.

The first disagreement consists in the inverted position of the retinal image; this is the ancient stumbling block of the theory of vision, and has occasioned the most different theories; among which the two following have gained the most adherents: According to one, conformably to the ideas of Jean Muller, it is necessary, in the notion which sight furnishes us, to consider the ideas of top and bottom as only relative, as indicating simply the relations between the positions of the different objects; we suppose then that the accord between the top and the bottom as we perceive them by touch and by sight, is attributable to the experience which we may acquire by looking at and touching at the same time. According to the second of these theories, we must admit, with L. Fick, that, in the brain, where they come necessarily to give rise to perceptions, the visual and sensitive fibres are grouped, in relation to each other, in a suitable manner to produce accord between the top and the bottom, the right and the left; an hypothesis which actually fails of the slightest anatomical foundation.

The second theory which the naturalistic theories encounter is, that we see single in spite of the presence of two retinal images. This difficulty is avoided by supposing that the two retinas, simultaneously excited; convey to the brain only one single sensation, the *corresponding* or *identical* points of the two retinas being coupled two and two. At first sight, this hypothesis seems to rest on an anatomical basis: the two optic nerves cross and penetrate reciprocally each into the other, before losing themselves in the brain. Moreover, certain pathological facts seem to prove that the fibres of the right halves of the two retinas end in the right cerebral hemisphere, and those of the left halves in the left hemisphere. But the interlacing of the corresponding fibres is a different thing from their fusion, and we know no anatomical fact which proves that the fibres unite in the same manner as the nerve trunks.

The two difficulties which we have just noted do not exist in the *impirical theory*, for which it suffices that the sensual sign, simple and compound, be recognized as a sign of the real object. He who is ignorant of the existence of the two retinas, of the inverted images which they receive, who has heard neither of the nerve fibres, nor of the transmission of the sensation to the brain, does not doubt the accuracy of his visual perceptions. The existence of two retinal images and their inversion matter little to him. He knows the impressions which the eye transmits to him in the presence of such or such an object situated in such or such a way, and this is sufficient for him. Now, two circumstrnces enable us to learn the signification of the local signs as it relates to our visual sensations; on the one hand, as we see the movable parts of our body, it is possible for us, after having acquired by touch the notions of space and of motion, to learn what are the modifications which such or such a movement of the hand may produce in our visual impressions; on the hand, when we move the eye, and consequently the retina, this membrance changes its place in relation to the retinal image which remains almost motionless, and in this manner we learn the impression which the same object produces upon different parts of the retina. The unchanging retinal image, whose position upon the retina is changed by the different motions of the eye, behaves like a pair of dividers which we apply to a design to learn the equality or inequality of the different distances which we find on it. Even when, which I am far from considering as probable, the local signs might be scattered without order or system, the proceeding which we have just indicated would enable us to recognize which those are that correspond, and what, upon the different regions of the retina, are the lengths which correspond to equal distances in the field of vision.

In keeping with this hypothesis, the experiments of Fechner, of Volkman and my own have shown that even as it regards the adult and perfectly formed eye, the comparision of distances and of angles is only made with accuracy, in the visual field, in the case where the normal movements of the eyes enable us to bring these distances or these angles so that they occupy successively the same line or the same superficial angle of the retina.

The experiment which I am about to describe enables us, moreover, to show how much, even in the adult, the accord between the tactile and visual impressions rests upon a constant verification which the vision gives to our hands. Let us put on some spectacles furnished with prismatic glasses, if the refracting angles of these prisms are situated vertically and to the right; the effect of the glasses is that all objects appear displaced toward the right. After having looked at an object through these spectacles without having allowed the hands to appear in the visual field, if we close the eyes and try to grasp the object, we pass invariably to the right of it. But if leaving the eyes open, we carry the hand slowly towards the object, we are no longer deceived; it is easy to move the hand so that its image comes and touches that of the point which we wish to attain. Handling the object for a minute or two without losing it from sight suffices to establish between the vision and the hand so perfect an accord that we no longer deceive ourselves when we wish to grasp the objects after having closed our eyes. More also, we no longer deceive ourselves if we wish to use the other hand which has not yet been employed in the experiment. It is therefore not the touch which has put itself into accord with these false images furnished by the vision; it is the visual perception which has adapted itself to the tactile perception and allows itself to be rectified by it. If we remove the glasses some time without looking at the hands, and if we close the eyes some instants later, the hands deceives itself a new and passes this time to the left of the objects; the relation which was established between the tactile and visual perceptions continues its effect, while we have returned things to their normal condition.

It is sufficient to make preparations under a compound microscope, which inverts the images, or even to shave one's self before a glass, to learn to direct our movements according to a visual impression different from that to which we are accustomed.

